# MiR-145 reprogramming facilitates fibrotic trajectory in the tumor microenvironment

**DOI:** 10.3389/fcell.2026.1850756

**Published:** 2026-10-19

**Authors:** Zhao Lin, Megan Bennett, Patrick Tassone, Joseph M. Curry, Ubaldo Martinez-Outschoorn

**Affiliations:** 1 Department of Medical Oncology, Sidney Kimmel Comprehensive Cancer Center, Thomas Jefferson University, Philadelphia, PA, United States; 2 Department of Otolaryngology, Sidney Kimmel Comprehensive Cancer Center, Thomas Jefferson University, Philadelphia, PA, United States

**Keywords:** CAF, EMT, hypomethylation, immune evasion, MiR-145, NOTCH signaling, TME

## Abstract

The fibrotic subtype of the tumor microenvironment characterized by epithelial-mesenchymal transition, fibrosis, angiogenesis and immune exclusion, predicts poor outcomes across diverse malignancies, yet the role of miR-145 in this context remains unexplored. Leveraging The Cancer Genome Atlas head and neck squamous cell carcinoma (HNSCC) and breast cancer (BRCA) databases, we demonstrate that miR-145 reprogramming recapitulates a noninflamed fibrotic phenotype with immune exclusion. In high miR-145 tumors, EMT programs persist alongside abundant cancer-associated fibroblasts, matrix remodeling and angiogenesis, with upregulation of NOTCH, TGF-β, myogenesis, hypoxia, Hedgehog, and Wnt/β-catenin signaling and downregulation of interferon-γ and JAK–STAT pathways. Our findings illustrate that cancer–stromal juxtacrine interactions activate NOTCH signaling, forming a positive feedback loop that amplifies miR-145 expression and is reinforced by miR-145 promoter hypomethylation. High miR-145 promotes autophagy and mitochondrial oxidative phosphorylation (OXPHOS) metabolism in cancer cells, with GAPDH serving as a central hub linking metabolism, noninflamed immune modulation, and poor clinical outcomes. *In vivo*, high miR-145 carcinoma cells co-engrafted with fibroblasts drive tumor growth through enhanced metabolism, angiogenesis and immune evasion. This study establishes miR-145 as a driver and biomarker of the fibrotic tumor microenvironment to better inform future cancer therapy strategies.

## Highlights

- miR-145 promotes epithelial-mesenchymal transition (EMT), NOTCH, angiogenesis, fibrosis, and TGF-β signaling in the tumor microenvironment (TME).
- miR-145 facilitates cancer immune evasion with deficient antigen processing and presentation (APP) and tumor immune exclusion.
- High miR-145 is a marker of fibrotic cancer subtype and predicts poor outcomes.

## Introduction

The tumor microenvironment is a key determinant of clinical outcome and therapeutic response, making TME-based classifications a promising source of novel drivers and biomarkers. TME comprises fibroblasts, endothelial cells, pericytes, mesenchymal stem cells, immune cells, and extracellular matrix components surrounding carcinoma cells. Based on immune and stromal characteristics, tumors can be classified into four TME subtypes: immune-enriched, non-fibrotic (IE); immune-enriched, fibrotic (IE/F); fibrotic (F); and immune-depleted (D) (Bagaev et al., 2021). The immune-favorable IE and IE/F subtypes show the greatest benefit from immunotherapy, whereas the F and D subtypes suppress anti-tumor immune infiltration and are associated with worse outcomes (Bagaev et al., 2021). To date, the connection between miR-145 and TME subtypes has not been addressed.

Immune evasion in cancer is linked to adverse outcomes. Aggressive tumors evade immune surveillance by recruiting immunosuppressive cells, including regulatory T cells (Tregs), myeloid derived suppressor cells (MDSCs), and tumor-associated macrophages (TAMs), while limiting infiltration and activity of effector immune cells. MDSCs play a central role in immune escape by suppressing cytotoxic T lymphocytes (CTLs), Natural Killer (NK) cells, and dendritic cells (DCs) through factors such as TGF-β, CCL2/5, CXCL2, IL-6/10, and VEGF. EMT and its transcription factors can induce cancer immune evasion (Imodoye and Adedokun, 2023). Developmental pathways such as TGF-β, NOTCH and Wnt signaling are involved in EMT programs in cancer (Sabit et al., 2025; Pelullo et al., 2019). EMT promotes immune evasion by disrupting immune synapse formation through E-cadherin loss, impairing antigen presentation, inducing autophagy-mediated resistance to CTL killing, and upregulating immune checkpoints (PD-L1, PD-L2, CTLA-4). EMT also enhances cancer stem cell traits, reduces CD8^+^ T-cell infiltration, and promotes recruitment of immunosuppressive cells (M Mullins et al., 2022; Hou et al., 2019). Recent spatial and single-cell transcriptomic studies in HNSCC identified a CAF subset expressing high levels of CXCL chemokines (CXCL9/10/12), MHC-I molecules, and Galectin-9 that suppresses anti-tumor immunity by promoting CD8^+^ T-cell dysfunction and exclusion (Li et al., 2024). In oral squamous cell carcinoma, FAP^+^ fibroblasts further drive immune evasion through WNT2-mediated β-catenin activation in macrophages, inducing a tumor-promoting C1QC^+^ phenotype that promotes T-cell exhaustion and resistance to anti-PD-1 therapy (Chen et al., 2026). Although miR-145 regulates multiple oncogenic pathways (Li et al., 2025; Xu et al., 2019; Cho et al., 2023; Johannessen et al., 2017; González-Cantó et al., 2024; Zeng et al., 2021), its specific role in mediating immune evasion remains undefined.

MicroRNAs (miRNAs) are small non-coding RNAs that regulate gene expression post-transcriptionally and play crucial role in tumorigenesis and cancer progression (Peng and Croce, 2016; Markopoulos et al., 2017; Wu et al., 2025). While most studies have focused on miR-145 in carcinoma cells, its effects on cancer aggressiveness remain controversial (Xu et al., 2019; Cho et al., 2023; Johannessen et al., 2017; González-Cantó et al., 2024; Zeng et al., 2021). Emerging evidence highlights important functions of miR-145 in non-cancer cells within the tumor microenvironment. Chivukula et al. reported that miR-143/145 expression is largely restricted to intestinal mesenchymal cells, including smooth muscle and pericryptal myofibroblasts (Chivukula et al., 2014). Similarly, Dimitrova et al. found that miR-145 is enriched in endothelial cells in lung cancer, where it promotes neoangiogenesis through Camk1d (Dimitrova et al., 2016). Given that miRNA expression is highly cell type–specific, its role in heterogeneous tumors should be interpreted in the context of both cancer and non-cancer cellular components of the TME.

We have focused on miR-145 in the TME of HNSCC and BRCA due to its dysregulation and shared TME features, including EMT, NOTCH signaling, angiogenesis, fibrosis, and immune exclusion. This study reveals that miR-145 expression is highest in the fibrotic TME subtype, highlighting the importance of understanding its regulatory mechanisms within the TME to inform cancer therapy strategies.

## Materials and methods

### Cell culture and treatment

Human breast carcinoma cell lines (MCF7, MDA231) and head and neck squamous carcinoma cell lines (FaDu, Detroit562) were obtained from ATCC. hTERT-immortalized human skin fibroblasts (hTERT-BJ1) were purchased from Clontech and engineered to express green fluorescent protein (GFP) or red fluorescent protein (RFP). Primary mouse embryonic fibroblasts (MEFs) were isolated from mouse embryo stroma. Cells were cultured in DMEM or EMEM supplemented with 10% fetal bovine serum, 100 U/mL penicillin, and 100 U/mL streptomycin. The mouse carcinoma cell line MOC1 was kindly provided by Dr. Ravi Uppaluri. For demethylation studies, cells were treated with 3 μM of 5-Aza-2′-deoxycytidine (5-Aza; A3656, Sigma) for 96 h.

### Co-culture and cell sorting

Fibroblasts and cancer cells were co-cultured at a 4:1 ratio, with a total of 1 × 10^5^ cells seeded per well in 12-well plates. As controls, fibroblasts or carcinoma cells were cultured alone at the same total cell number. For cell separation, GFP- or RFP-expressing fibroblasts and cancer cells were cultured either in co-culture or homotypic culture in 10-cm dishes for 4 days, followed by fluorescence-activated cell sorting (FACS) performed by the Flow Cytometry Core Facility at the Sidney Kimmel Comprehensive Cancer Center at Thomas Jefferson University. Sorted cells were collected and used for subsequent analyses.

### Generation of stable cell lines

Lentiviral plasmids encoding precursor miR-145, scrambled control, NOTCH1ΔNT, and empty vector control were purchased from GeneCopoeia. Lentiviral production, transfection, and transduction were performed according to the manufacturer’s instructions to establish stable cell lines.

### Flow cytometry analysis

Cancer cells were co-cultured with BJ1 fibroblasts or maintained in homotypic culture. After 4 days, cells were processed at room temperature using immunofluorescence (IF) buffer (PBS, 1% BSA, 0.1% Tween-20). Cells were fixed with 4% paraformaldehyde for 15 min, blocked with IF buffer containing 5% normal goat serum for 30 min, and incubated with anti-NOTCH1 primary antibody for 1 h followed by fluorochrome-conjugated secondary antibody for 30 min. After washing with IF buffer, cells were resuspended in PBS and analyzed by flow cytometry.

MitoTracker Orange (M7510), LysoTracker Blue (L7525), and LysoSensor Blue (L7533) (Invitrogen) staining was performed according to the manufacturer’s instructions and quantified by flow cytometry.

### DNA methylation analysis

CpG islands were predicted using the CpGPlot in the EMBOSS from EMBL-EBI. DNA was isolated from cell lines using FitAmp General Tissue DNA Isolation Kit (P-1003-1, Epigentek). DNA Methylation at premiR-145 promoter region containing CpG rich islands were screened using OneStep qMethyl™kit (D5310, ZYMO Research). Primer sequences were as follows:

premiR-145Me-F1CAGGCTAGGAACTGAATGGTT
premiR-145Me-R1CTGGAGTTCTCTTCTGCATCC
premiR-145Me-F2GCACCAGTTCTCATGTGGAT
premiR-145Me-R2AGTTCTGCAGGTCTGTTTAGTG

### MiR array

Cell culture media was collected, and total RNA was isolated using exoEasy Maxi and RNeasy Plus kits (Qiagen). MiRNA array was performed at MetaOmics Core Facility at the Sidney Kimmel Comprehensive Cancer Center at Thomas Jefferson University.

### Quantitative RT-PCR

Cells were harvested and total RNA was isolated using trizol (invitrogen). TaqMan miRNA assays (Applied Biosystems) were used to quantify the expression of miRNAs according to the manufacturer’s instructions. The mean cycle threshold (CT) value was determined from three PCR replicates. Data was reported using the 2-ΔΔCt method. Experiments were normalized to RNU48.

### Proteomic assay

All MS runs were performed at Applied Biomics (www.appliedbiomics.com) for Mass and Proteomics Spectrometry. Protocol details have been previously published extensively (Lin et al., 2024).

### Antibody

NOTCH1(ab52627, abcam), cyclin-D1(NBP1-35732, Novus Biologicals), SQSTM1(18420-1-AP, proteintech), COMMD1 (NBP2-4633, Novusbio), P53 (SC-126, Santa Cruz Biotechnology), DNMT1(5032, Cell Signaling),CD8A (98941, Cell Signaling), Jagged1 (70109, Cell Signaling), VEGFR2 (9698, Cell Signaling), B-actin (A5441, Sigma), and tubulin (T4026, Sigma) antibodies were used.

### OCR assessment

A Seahorse Bioscience XF24 Extracellular Flux Analyzer in a 24-well format was used. MOC1 cells were seeded in the 24-well plate with 100 μL of growth media. After 2 h, additional 200 μL of growth medium was added to each well. On the second day, cells were incubated in non-buffered seahorse media containing 5 mM glucose and 10 mm lactate in a CO_2_ free incubator for 2 h. Cells were subjected to the mito stress test according to the manufacturer’s manual.

### Glucose assay

Intracellular Glucose was measured using the Glucose (GO) Assay Kit (Sigma, GAGO20-1KT) according to the manufacturer’s manual.

### ATP assay

Intracellular ATP level was measured using the ATP Determination Kit (ThermoFisher A22066) according to the manufacturer’s manual.

### IHC assay

Mouse tumor paraffin sections were de-paraffinized and rehydrated. Antigen retrieval was performed in 10 mM citrate buffer pH 6.0 for 10 min in a pressure cooker. Each section was first blocked with 3% H_2_O_2_ for endogenous peroxidase for 15 min, second with an avidin-biotin blocking kit for endogenous biotin, third with preferred blocking solution for 1 h at room temperature. Then incubated with primary antibody overnight at 4 °C and with secondary antibody for 1 h, followed by an avidin-biotinylated horseradish peroxidase complex. Immune-complexes were visualized using the Opti-View DAB IHC Detection Kit and Opti-View Amplification Kit. Lastly, all sections were counterstained with hematoxylin and sealed with mounting medium.

### Animal studies

All animal experiments were performed in compliance with Thomas Jefferson University Institutional Animal Care and Use Committee (IACUC) policies.

To evaluate the *in vivo* tumor-promoting effects of miR-145, Detroit562 expressing either precursor miR-145 or scramble control, and hTERT-BJ1cells were coinjected into the dorsal subcutaneous flank of nude mice (NCRNU; Taconic Farms; at 6 weeks of age). Cancer cells (1 million cells) and hTERT-BJ1 (0.3 million) fibroblasts were resuspended in 100ul of sterile PBS just before injection. Tumors were measured at the chasing time. After 28 days post-injection, tumors were excised to determine their size. Volumes were calculated using the ellipsoid formula (X^2^Y)/2 where X is the width or short axis and Y is the length or long axis. P values less than 0.05 were considered significant based on the Mann-Whitney U test.

In addition, MOC1 expressing either precursor miR-145 or scramble control, and MEF cells were coinjected into the dorsal subcutaneous flank of female mice (C57BL/6; Jackson lab; at 6 weeks of age). Cancer cells (1 million) and MEF fibroblasts (1 million) were resuspended in 100 ul of sterile PBS just before injection. Tumors were measured at the chasing time. The growth of tumor xenografts was assessed at 21 days.

### Statistical analysis

Data was expressed as means ± standard error of the mean. One-way or two-way analysis of variance (ANOVA) was applied to grouped analyses using Prism 10 software (GraphPad Software Inc., La Jolla, CA). The unpaired Mann-Whitney U test was used for statistical comparison *in vivo*. The p-values less than 0.05 were set to define statistical significance. R (http://www.R-project.org) was adopted to analyze miRNAseq and RNAseq data in The Cancer Genome Atlas (TCGA) breast cancer (BRCA) and head and neck squamous cell carcinoma (HNSCC) databases. Statistical significance was defined as p-values of less than 0.05.

## Results

### High miR-145 expression correlates with the fibrotic subtypes in human HNSCC and BRCA

To investigate the role of miR-145, miRNA-seq and RNA-seq data from TCGA HNSCC database was retrieved and classified into four distinct TME subtypes: IE, IE/F, F, and D. Overall survival analysis showed that the F subtype had the poorest prognosis (Figure 1A). Consistently, miR-145 expression was highest in F tumors (Figure 1B). Gene Set Variation Analysis (GSVA) of Hallmark pathways revealed positive correlations between miR-145 and EMT, angiogenesis, NOTCH, hypoxia, TGF-β, Hedgehog, myogenesis, and Wnt/β-catenin signaling, and negative correlations with OXPHOS and IFN-γ pathways (Supplementary Figures 1A–C). MiR-145 expression was consistent with the contingent Hallmark pathways in the F subtype (Figures 1C,D; Supplementary Figure 1D). Also, miR-145 expression was correlated with a panel of genes from diverse signaling pathways such as angiogenesis, EMT and immunosuppression. In addition, miR-145 expression positively correlated with markers of CAFs (ACTA2, COL1A1), angiogenesis (PECAM1, ANGPT1), EMT transcription factors (SNAI1, TWIST1, ZEB1), and TGF-β signaling (TGFB2, TGFB3), while negatively correlating with the immune marker CD8A (Supplementary Figures 2A–C). Together, these findings suggest that miR-145 may serve as a biomarker of the fibrotic subtype.

**FIGURE 1:**
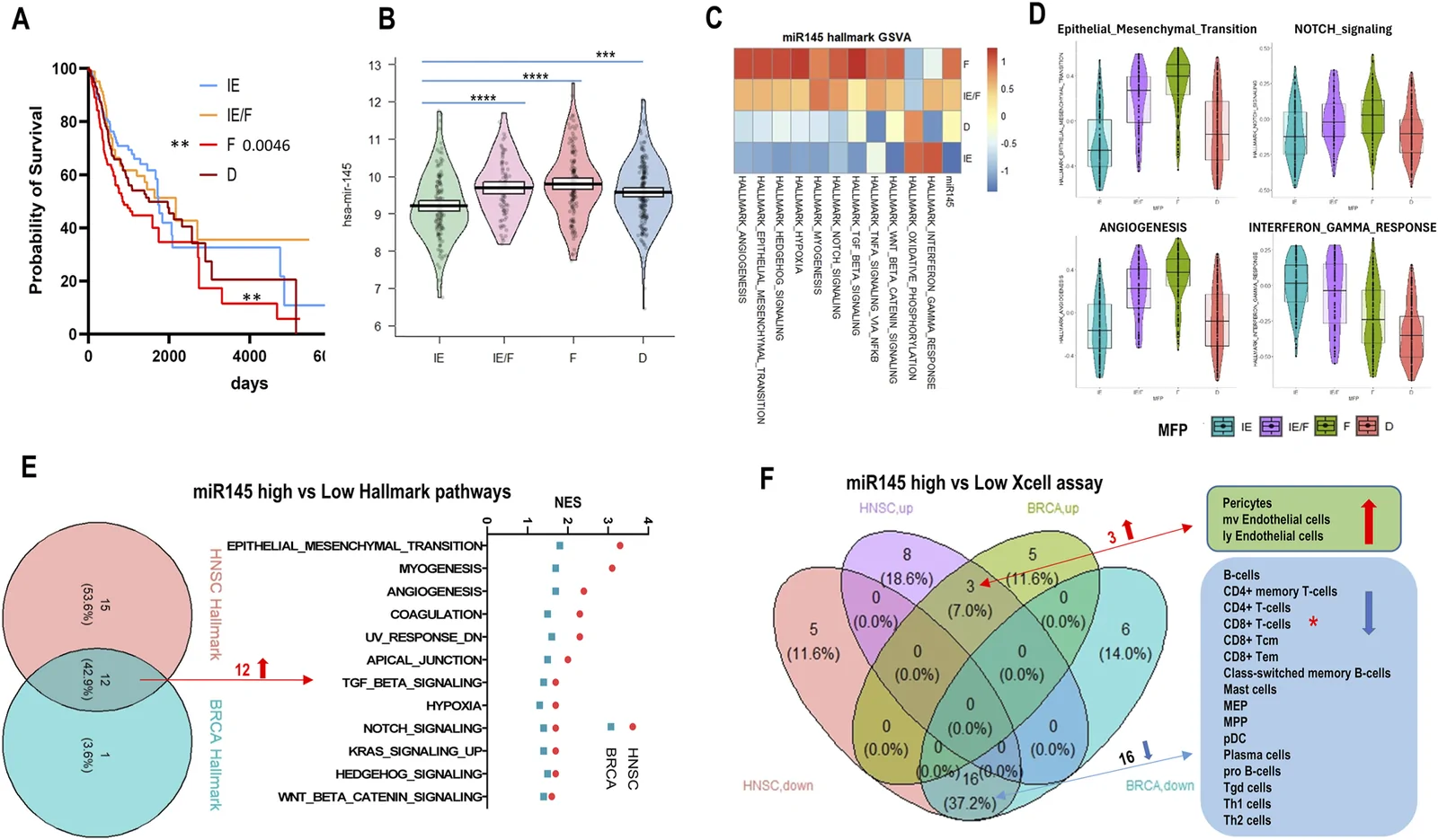
High MiR-145 expression facilitates a fibrotic TME subtype in human HNSCC and BRCA. **(A–D)** TCGA HNSC miRNA-seq and RNA-seq data were classified into four TME subtypes (IE, IE/F, F, and D). **(A)** Overall survival by subtype. **(B)** miR-145 expression across subtypes. **(C)** Heatmap of miR-145 expression and GSVA Hallmark pathways (EMT, angiogenesis, NOTCH, hypoxia, TGF-β, Hedgehog, myogenesis, WNT/β-catenin, OXPHOS, and IFN-γ). **(D)** Violin plots showing EMT, angiogenesis, NOTCH, and IFN-γ pathway enrichment across subtypes. **(E,F)** TCGA HNSCC and BRCA miRNA-seq and RNA-seq data were stratified into the top 25% high and low miR-145 expression groups. **(E)** GSEA of Hallmark pathways enriched in high miR-145 tumors in both HNSCC and BRCA. **(F)** Venn diagram of xCell analysis.

TCGA HNSCC and BRCA miRNA-seq/RNA-seq datasets were stratified into the top and bottom 25% of miR-145 expression. Gene Set Enrichment Analysis (GSEA) identified 12 Hallmark, 37 Reactome, and 322 Gene Ontology (GO) pathways significantly enriched in miR-145-high tumors across both cancer types (Figure 1E; Supplementary Figures 3A,B). Hallmark pathways included EMT, angiogenesis, NOTCH, hypoxia, TGFβ, HEDGEHOG, Wnt/β-catenin, and myogenesis signaling, consistent with TME reprogramming (Figure 1E). Reactome analysis highlighted extracellular matrix (ECM) remodeling pathways, including ECM organization, proteoglycan and collagen formation, integrin and laminin interactions, matrix metalloproteinase activation, and ECM degradation (Supplementary Figure 3A). Gene Ontology analysis further revealed enrichment of angiogenic and lymphangiogenic programs, including sprouting angiogenesis, vasculogenesis, endothelial cell migration and proliferation, VEGF and PDGFR signaling, TGFβ responses, growth factor activity, integrin and proteoglycan binding, Wnt signaling, lymphatic vessel development, and SMAD-mediated signaling (Supplementary Figure 3B). Together, these findings indicate that high miR-145 expression is associated with extensive ECM remodeling and enhanced angiogenic/lymphangiogenic TME programs.

Deconvolution of TME cell types using xCell analyses revealed that high miR-145 expression was associated with increased endothelial cells and decreased immune cell infiltration in both HNSCC and BRCA (Figure 1F). Innate immune populations, including mast cells and plasmacytoid dendritic cells (pDCs), were reduced, with more pronounced depletion of adaptive immune cells, particularly B and T lymphocytes. Mast cells in the TME can exert either tumor-promoting or tumor-suppressive effects. Their anti-tumor activity includes releasing cytotoxic cytokines such as TNFα and recruiting dendritic cells and effector T Cells through chemokines. Similarly, pDCs are rare TME residents that undergo functional reprogramming. Although healthy pDCs promote antitumor immunity through robust production of type I interferons (IFN-α/β), they can also acquire protumorigenic functions within the TME. Consistent with this immunosuppressive phenotype, OncoLnc analysis showed significantly worse overall survival in HNSCC and BRCA patients with low expression of the cytotoxic T-cell marker CD8A (Supplementary Figure 3C).

To further unravel the fibrotic landscape of miR-145, using single-sample GSEA (ssGSEA) of the TCGA HNSCC RNA-seq dataset, we found that tumors with high miR-145 expression displayed a highly fibrotic, immune-excluded phenotype. These tumors were enriched for angiogenesis, CAFs and extracellular matrix remodeling (Figure 2A), consistent with elevated TGF-β signaling. Malignant cells exhibited enhanced EMT and immune-evasive features, including increased MDSC recruitment, reduced antigen processing and presentation (APP), and diminished infiltration of effector immune cells, as reflected by lower M1 macrophage, Th1, NK cell, and CD8^+^ T-cell signatures (Figures 2B–G). This immune-suppressive phenotype was further supported by downregulation of the JAK–STAT signaling pathway (Figure 2H).

**FIGURE 2:**
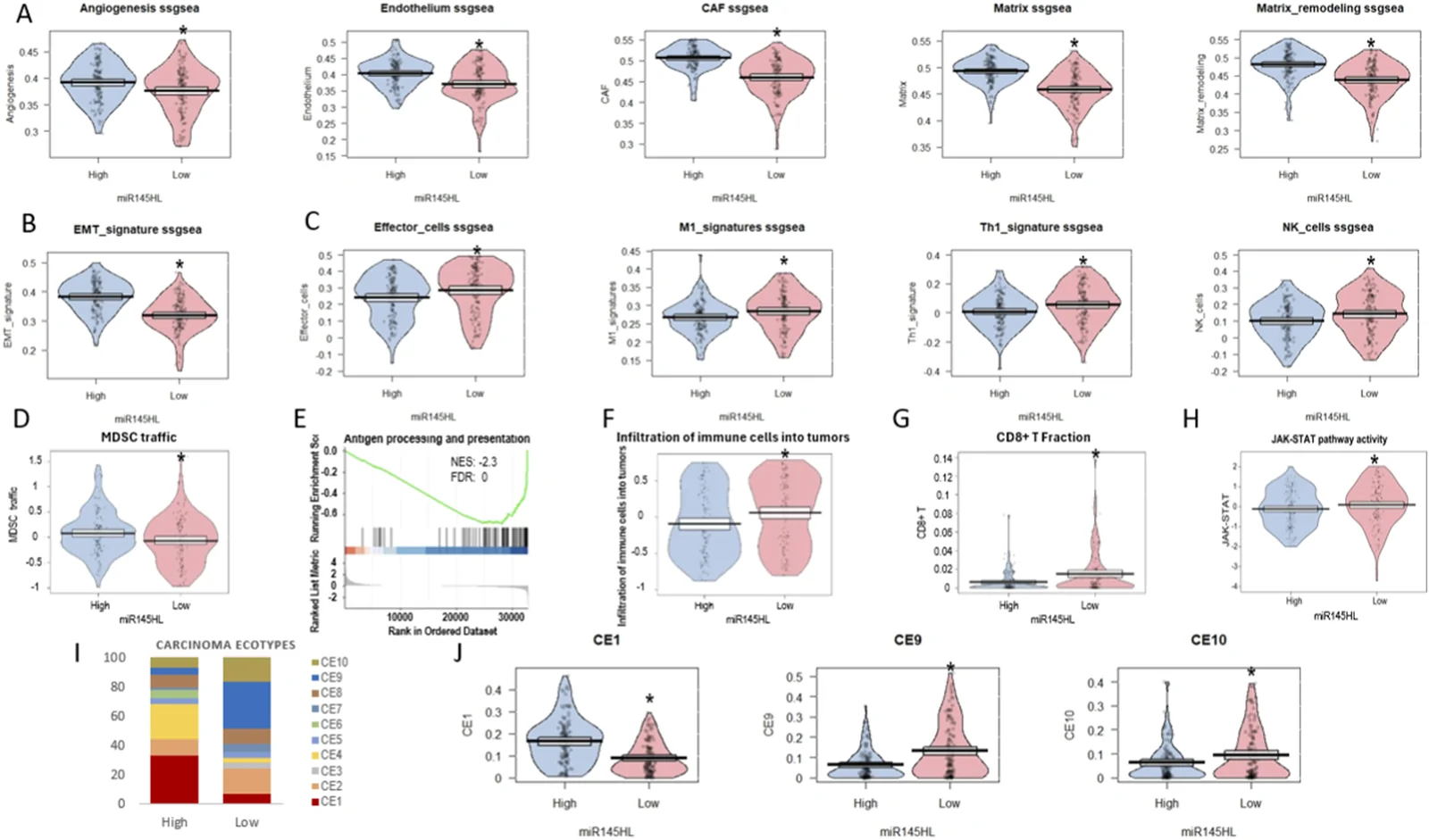
High miR-145 in human HNSCC curated gene sets to develop a fibrotic phenotype. **(A–J)** TCGA HNSC miRNA-seq and RNA-seq data were stratified into high and low miR-145 expression groups (top 25% vs. bottom 25%). High versus low group displays with adjusted p < 0.05. **(A–C)** ssGSEA was based on 28 gene signatures reference. **(A)** Angiogenesis and fibrosis, **(B)** EMT, and antitumor microenvironment signatures **(C)**. **(D–H)** IOBR analysis of tumor immunity, including MDSC trafficking **(D)**, antigen processing and presentation **(E)**, immune cell infiltration **(F)**, CD8 T-cell fraction **(G)**, and JAK-STAT pathway activity **(H)**. **(I,J)** Distribution and abundance of carcinoma ecotypes (CE1, CE9, and CE10) in high and low miR-145 groups.

Ecotyper deconvolution allowed us to define carcinoma cell states and ecotypes (Luca et al., 2021). MiR-145–high tumors were enriched for Carcinoma Ecotype 1 (CE1) and depleted for CE9 and CE10 ecotypes (Figures 2I, J). Importantly, CE1 is associated with poor prognosis, low lymphocyte infiltration, and abundant periostin positive (POSTN+) fibroblasts, whereas CE9 and CE10 are linked to improved survival and enhanced immune activity (Luca et al., 2021). Consistent with CE1, miR-145–high tumors were enriched for POSTN + fibroblasts (state 3-S03), partial EMT-like epithelial cells (state 3-S03), and ANGPTL2+/NID2+ tip-like endothelial cells (state 2-S02), all associated with tumor progression and neovascularization (Supplementary Figures 4A–C). In contrast, cytotoxic GZMB + CD8 T cells (state 3-S03), GZMK + CD8 T cells (state 1-S01), CE9/CE10-associated T cells, and PRF1+ NK cells (state 1-S01) were reduced (Supplementary Figures 4D,E). Macrophage polarization also shifted toward an unfavorable phenotype, with decreased M1 macrophages (state 3-S03) and increased M2 macrophages (states 5 and 6-S05 and S06) (Supplementary Figures 4F). Together, these findings indicate that high miR-145 expression is associated with a non inflamed fibrotic tumor microenvironment.

### MiR-145 in cancer cells can be upregulated by fibroblasts

To investigate the mechanism of miR-145 expression, we established a coculture system of fibroblasts and carcinoma cells at a 4:1 ratio. After 4 days of homotypic culture or coculture, exosomes were isolated from conditioned media and analyzed by miRNA array. miR-145 was highly enriched in fibroblast-derived exosomes compared with those from MCF7 breast carcinoma cells under homotypic culture (Figure 3A). Consistently, intracellular miR-145 expression was markedly higher in fibroblasts (hTERT-BJ1) than in breast carcinoma cells (MCF7, MDA231) or head and neck squamous carcinoma cells (FaDu, Detroit562) (Figure 3B).

**FIGURE 3:**
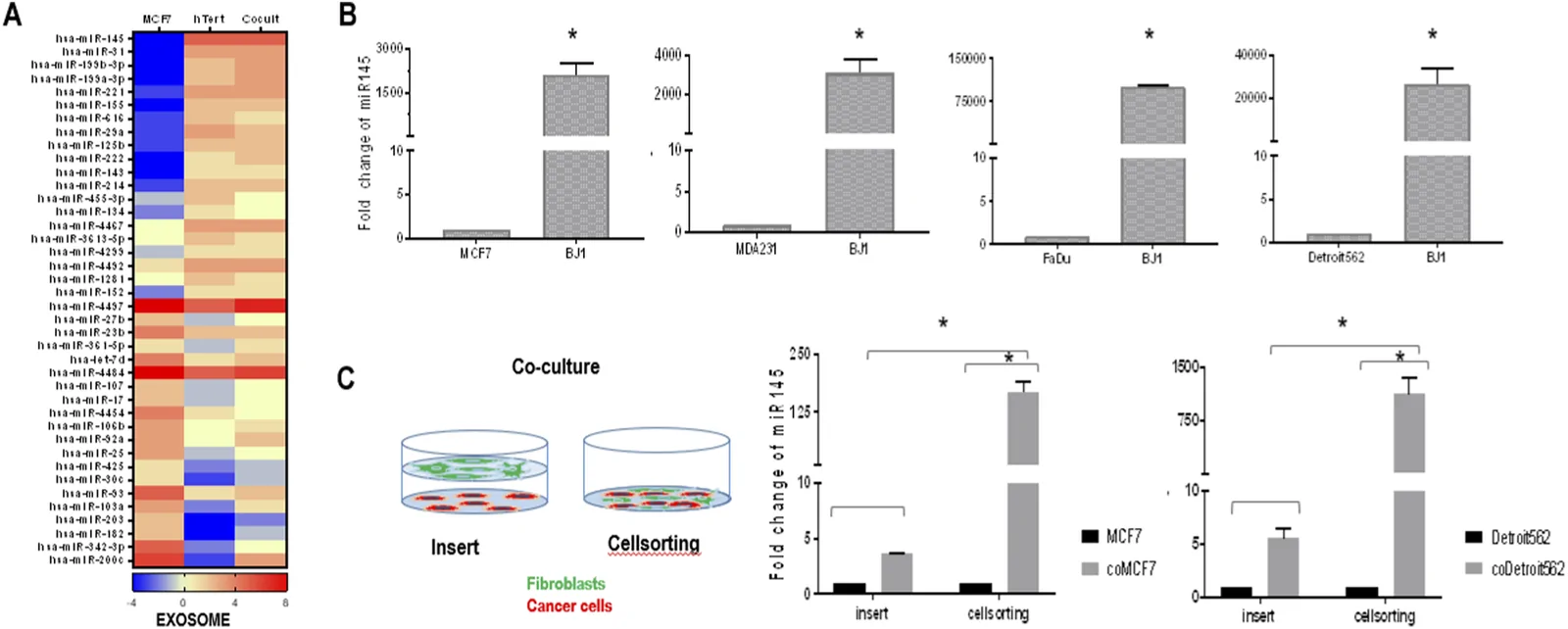
Specific miRNA signatures derived from cancer cells and fibroblasts. **(A)** miRNA array heatmap of exosomal miRNAs collected from homotypic and coculture conditions of hTERT-BJ1 fibroblasts and MCF7 cells after 4 days. **(B)** Fold change of miR-145 expression in hTERT-BJ1 fibroblasts and various cancer cells. **(C)** Fold change of miR-145 level in carcinoma cells cultured alone or with fibroblasts using insert and cell-sorting approaches (n = 3; p < 0.05). Data are shown as mean ± SD.

To assess the effect of fibroblasts on miR-145 expression in carcinoma cells, two coculture systems were used: a transwell setup with physical separation and a direct-contact coculture followed by GFP/RFP-based cell sorting. Only direct cell-cell juxtacrine interaction markedly induced miR-145 expression in carcinoma cells (Figure 3C).

### Global protein interaction network analysis and functional prediction

To Identify putative targets of miR-145 regulation in cancer cells, we performed a proteomic assay with Detroit562 cells ectopically expressing precursor miR-145 or scramble control. Twenty-one proteins showing >1.5-fold differential expression were identified as candidate miR-145-regulated targets (Figure 4A). Among them, phosphoserine phosphatase (PSPH) was the most upregulated protein in miR-145-overexpressing cells. PSPH is a key enzyme in the serine biosynthesis pathway and is metabolically linked to glycolysis. Notably, elevated PSPH expressions have been associated with poor prognosis in HNSCC. To investigate the biological significance of these proteins, all differentially expressed candidates were analyzed using STRING to construct a protein–protein interaction (PPI) network and identify associated cellular functions (Figure 4B). The analysis revealed that glyceraldehyde-3-phosphate dehydrogenase (GAPDH) occupied the most connected node within the network. Subsequent focused analysis of GAPDH and its interacting partners highlighted cell death, proliferation, ATP production, and oxidative stress as the principal associated biological processes. Functional enrichment further identified glycolysis/glycogen metabolism, mitochondrial electron transport, and immune-related pathways, with oxidation- and methylation-related annotations also significantly represented. Importantly, elevated GAPDH expression was associated with reduced overall survival in the TCGA HNSCC cohort (p = 0.01; Figure 4C). High GAPDH expression is also linked to a non-inflamed tumor microenvironment including fibrotic and immune-depleted subtypes (Figure 4D).

**FIGURE 4:**
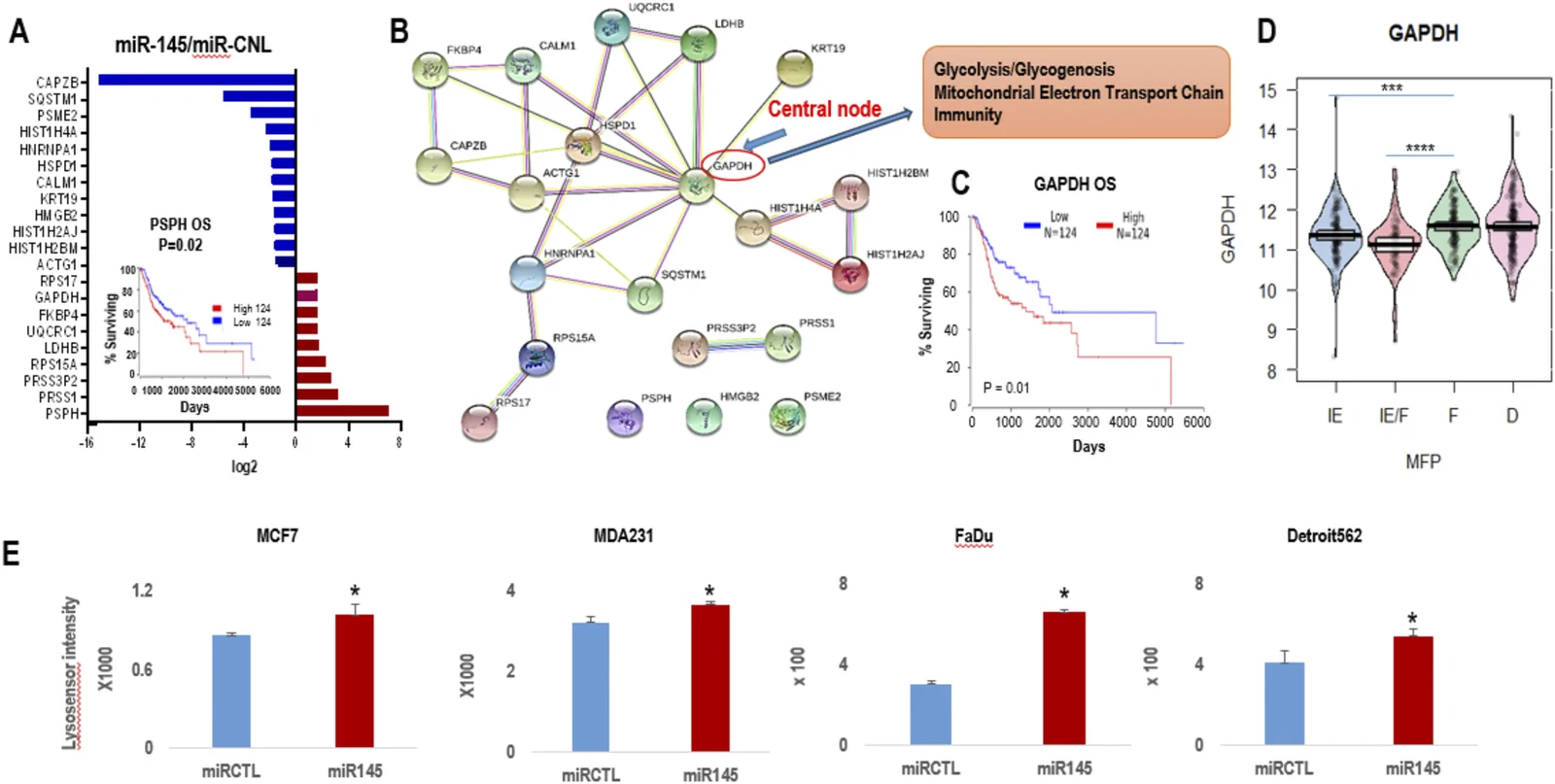
Global protein interaction network analysis and functional prediction. **(A,B)** Proteomic profiling of Detroit562 cells expressing precursor miR-145 or scramble control identified proteins with >1.5-fold change, visualized by bar plot and STRING network analysis. Overall Survival of PSPH Kaplan-Meier Estimate in TCGA HNSC. **(C)** Overall Survival of GAPDH Kaplan-Meier Estimate in TCGA HNSCC cancer database. **(D)** Association of elevated GAPDH expression with a non-inflamed TME in TCGA HNSC classified into four TME subtypes (IE, IE/F, F, and D). **(E)** Flow charts showing lysosensor intensity change in indicated cancer cells expressing precursor miR-145 and scramble control. (n = 3, asterisk indicates P < 0.05). Error bars denote ±SD.

Conversely, miR-145 may promote autophagy, as two autophagy-related proteins were downregulated upon miR-145 overexpression: the F-actin-capping protein CAPZB and SQSTM1 (Figure 4A). CAPZB mediates actin-dependent autophagosome formation, and SQSTM1 recruits ubiquitinated cargo as a classic selective autophagy receptor and a negative reporter of autophagy activity. Since lysosomes are essential for autophagic degradation and cellular adaptation to nutrient or oxygen deprivation, we evaluated lysosomal activity using LysoTracker and LysoSensor (pH sensor) assays. Multiple cancer cell lines overexpressing miR-145 showed increased LysoTracker and LysoSensor signals compared with controls, consistent with enhanced autophagy (Figure 4E; Supplementary Figures 5A,B).

### MiR-145 stimulated mitochondrial activity

Mitochondrial dysfunction is a hallmark of cancer and is linked to metastasis and drug resistance. Using the mitochondria-selective probe Mitotracker Orange, we found that miR-145 increased mitochondrial mass and membrane hyperpolarization in MCF7, MDA231, FaDu, and Detroit562 cells (Figure 5A). Consistently, ATP production and glucose uptake were also elevated following miR-145 overexpression (Figures 5B,C), indicating enhanced mitochondrial activity.

**FIGURE 5:**
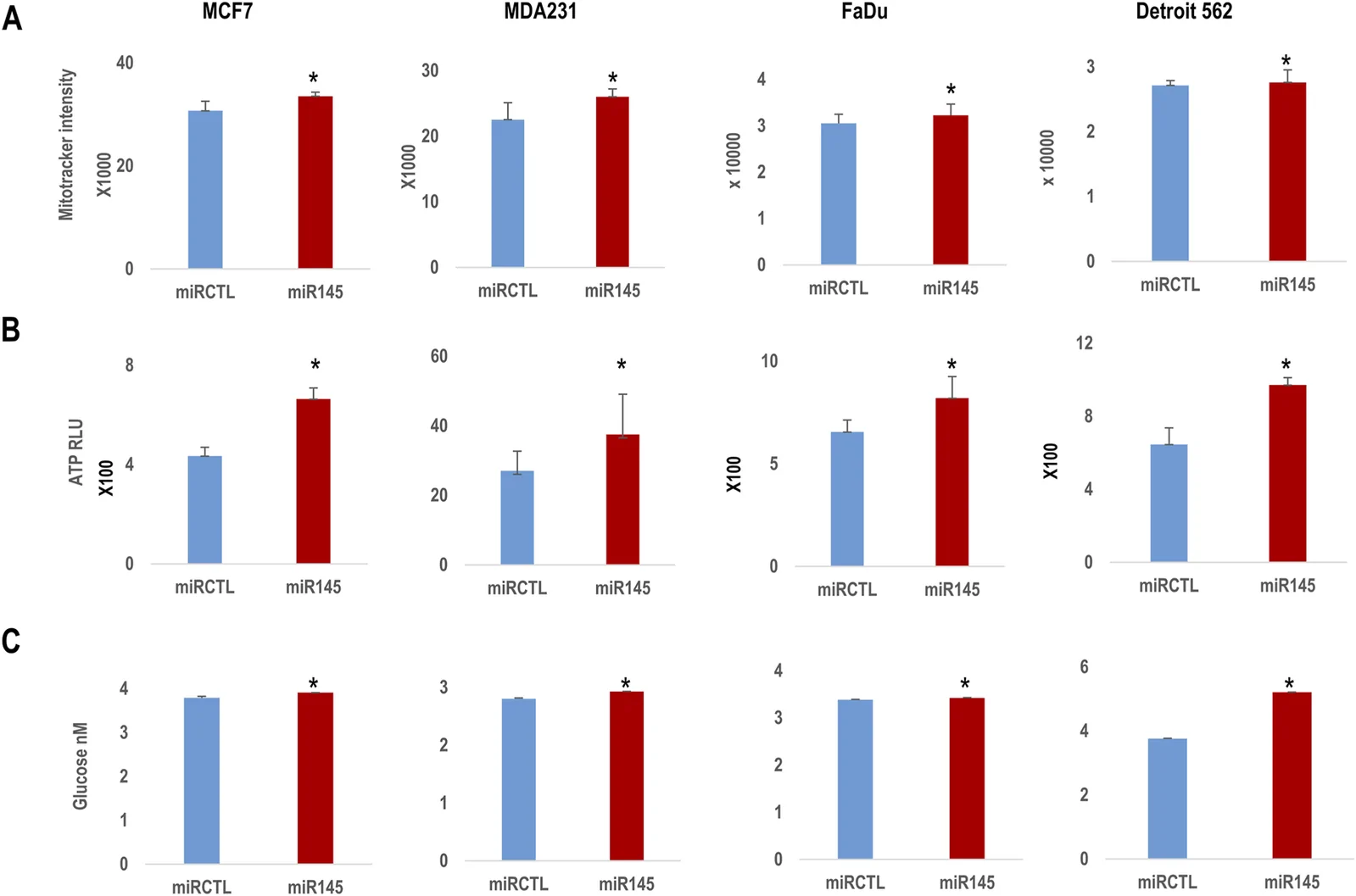
miR-145 stimulates mitochondrial activity. **(A)** Flow charts showing mitotracker intensity change in indicated cancer cells expressing precursor miR-145 and scramble control. **(B)** ATP level in various cancer cells expressing precursor miR-145 and scramble control. **(C)** Glucose uptake represented in different cancer cells expressing precursor miR-145 and scramble control.

### miR-145 promotes tumor growth with aggressive characteristics

To investigate the role of miR-145 in tumor metabolism, Seahorse XF Cell Mito Stress Tests were performed in MOC1 and Detroit562 cells. The assay reflects key parameters of mitochondrial function by simultaneously measuring the oxygen consumption rate (OCR) and extracellular acidification rate (ECAR) over time in the presence of mitochondrial metabolic modulators. OCR and ECAR rates are key indicators of mitochondrial respiration and glycolysis respectively. miR-145 overexpression increased both OCR and ECAR, indicating enhanced mitochondrial OXPHOS and glycolysis (Figures 6A,B; Supplementary Figures 5C).

**FIGURE 6:**
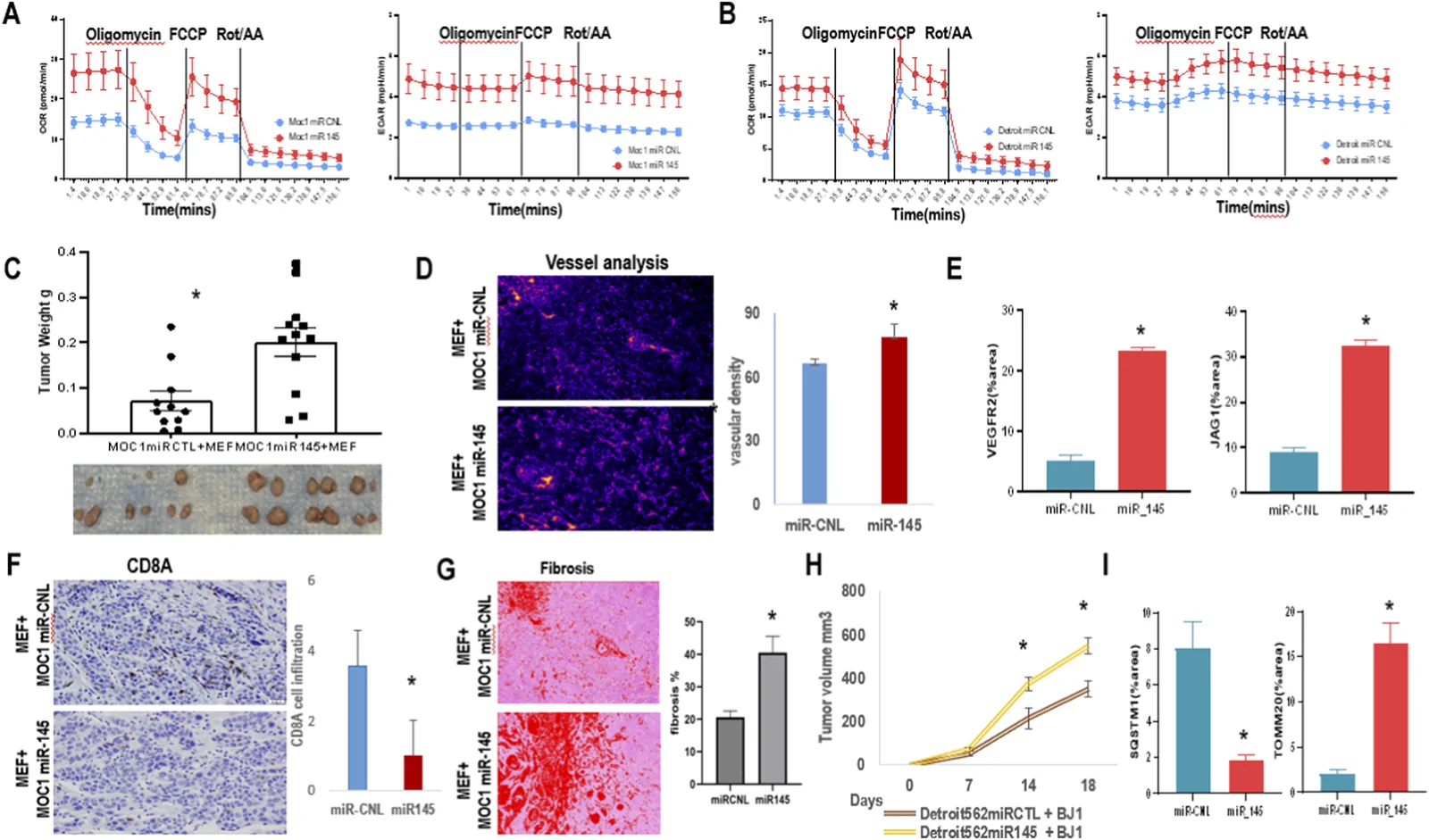
miR-145 promotes tumor growth with aggressive features. **(A,B)** OCR and ECAR measurements in MOC1 and Detroit562 cells expressing precursor miR-145 or scramble control to assess mitochondrial oxidative and glycolytic metabolism (p < 0.05). **(C–G)** Tumor growth of MOC1 cells *in vivo*. MEFs with MOC1 cells overexpressing miR-145 or scramble control were injected into mice. **(C)** Tumor weight at endpoint (4 weeks; n = 11–12; asterisk indicates P < 0.05). **(D)** Tumor vessel analysis by H&E staining. **(E)** Area fraction of IHC staining for angiogenesis marker VEGFR2 and Jagged1. **(F)** CD8A IHC staining. **(G)** Using ImageJ quantifies the fibrosis (collagen deposition) in H&E-stained image and calculates the percentage area. **(H,I)** Tumor growth of Detroit562 cells *in vivo*. hTERT-BJ1 fibroblasts with Detroit562 cells expressing miR-145 or scramble control were injected into mice. **(H)** Tumor volume was monitored over time (n = 10, asterisk indicates P < 0.05).

*In vivo*, miR-145-overexpressing MOC1 cells coinjected with MEF fibroblasts formed larger tumors than controls (Figure 6C). Vessel analysis using ImageJ from tumor tissue H&E staining revealed that miR-145 promoted tumor angiogenesis and lymphangiogenesis (Figure 6D; Supplementary Figure 6A). miR-145 also increased expression of VEGFR2 and Jagged1 (Figure 6E; Supplementary Figure 6B) and reduced CD8A^+^ cytotoxic T-cell infiltration (Figure 6F). We also observed significantly increased fibrosis in miR-145-overexpressing MOC1 tumors compared with controls (Figure 6G). Likewise, Detroit562 cells overexpressing miR-145 co-injected with BJ1 fibroblasts promoted tumor growth (Figure 6H; Supplementary Figure 6C) with suppressed SQSTM1 and induced TOMM20 expression (Figure 6I; Supplementary Figure 6D), consistent with elevated mitochondrial activity. Low SQSTM1 is associated with high autophagy but future studies are required to determine conclusively the role of miR-145 on autophagy *in vivo*. Collectively, these findings suggest that miR-145 promotes tumorigenesis through enhanced metabolism, angiogenesis, and immune evasion.

### MiR-145 is regulated by NOTCH signaling and DNA hypomethylation

The requirement for carcinoma–fibroblast juxtacrine interaction to induce miR-145 suggested involvement of membrane-bound NOTCH signaling. As expected, coculture markedly increased NOTCH1 expression in carcinoma cells, as shown by IF and flow cytometry (Figures 7A,B; Supplementary Figures 7A,B). Western blot showed that miR145 overexpression induced the functional, cleaved intracellular domain of the Notch1 receptor (NICD) (Figure 7C). In the TCGA-HNSC dataset, the fibrotic subtype exhibited the highest expression of both miR-145 and the NOTCH signaling pathway. Consistently, this subtype also showed the highest expression of the cell-surface Notch ligand JAG1 (Figure 7D), which initiates NOTCH signaling in neighboring cells. Moreover, NOTCH1ΔNT overexpression significantly elevated miR-145 levels across multiple cell lines compared with controls (Figure 7E; Supplementary Figure 7C). Together, these findings demonstrate a positive feedback loop between NOTCH signaling and miR-145.

**FIGURE 7:**
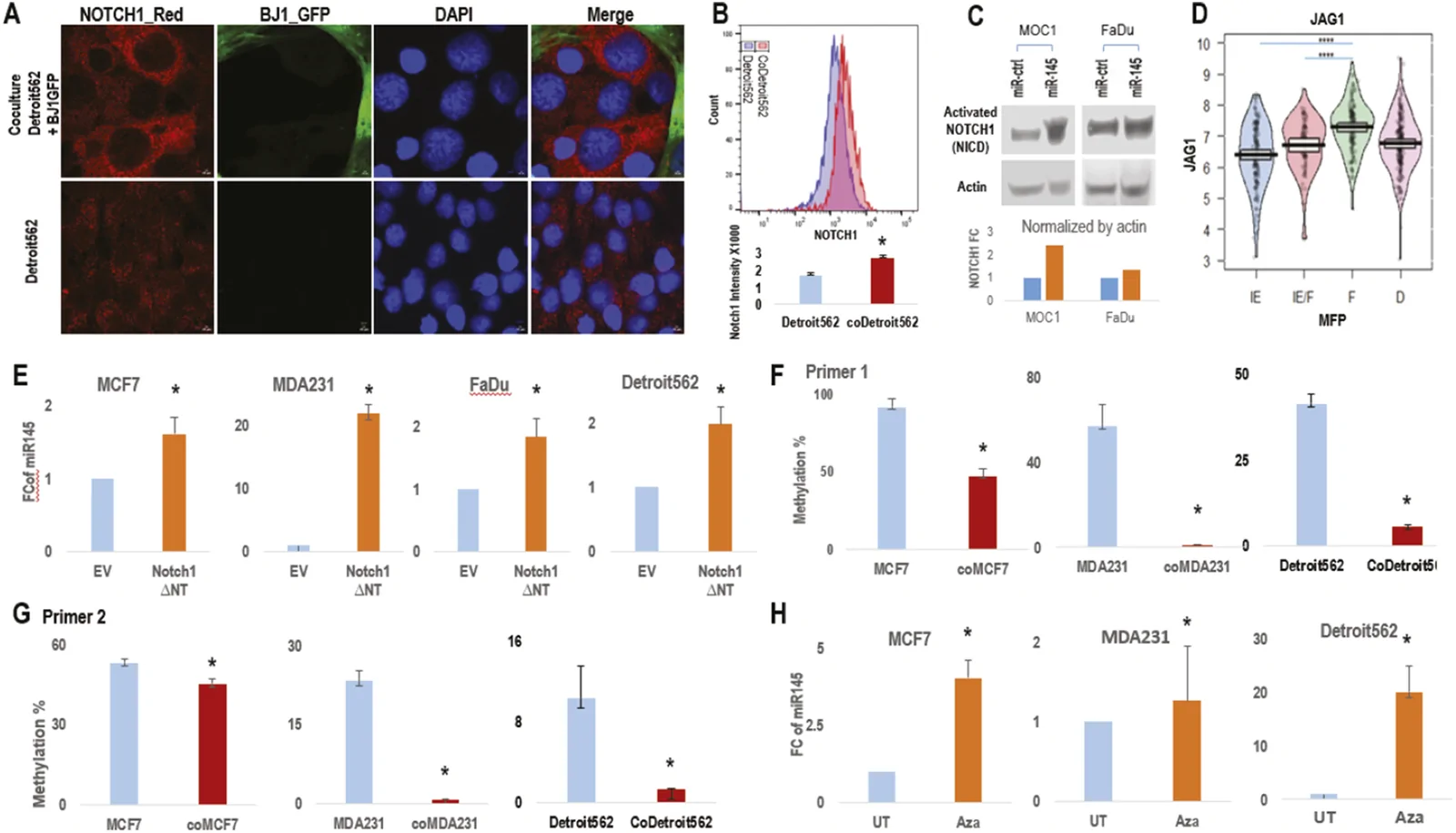
MiR-145 regulation and its impact on the TME. **(A)** Images of NOTCH1 (Red) expression in Detroit562 cancer cells under cocultured with BJ1GFP or homotypic culture condition (scale bar: 20 μm). **(B)** Flow plot showing NOTCH1 level in Detroit562 cancer cells under fibroblast coculture or homotypic culture conditions. (n = 3, asterisk indicates P < 0.05). Error bars denote ±SD. **(C)** Activated NOTCH1(NICD) expression in MOC1 and FaDu carcinoma cells overexpressing precursor miR145 and scramble control. **(D)** JAG1 expression in TCGA HNSC classified into four TME subtypes (IE, IE/F, F, and D). **(E)**Fold change of miR-145 expression in hTERT-BJ1 and cancer cells overexpressing NOTCH1ΔNT or control vector. **(F,G)** Promoter DNA methylation change of premiR-145 in BJ1 and various cancer cells. **(H)** Fold change of miR-145 expression in MCF7, MDA231 and Detroit562 cells after Aza treatment.

Epigenetic changes, particularly DNA methylation, occur early in tumor development and can regulate gene expression. To determine whether DNA methylation controls miR-145 expression, bioinformatics and methylation-specific PCR analyses were performed. miR-145 promoter methylation was markedly reduced in cocultured cancer cells compared with homotypic cultures (Figures 7F,G), while BJ1 cells exhibited lower methylation levels than carcinoma cells (Supplementary Figure 7D). Furthermore, treatment with the DNA methylation inhibitor azacitidine (5-Aza) significantly increased miR-145 expression in cancer cells (Figure 7H). Western blot showed increased TP53 and reduced DNMT1 and COMMD1 expression in hTert-Bj1 and MCF7 cells (Supplementary Figure 7E). p53 can promote miR-145 post-transcriptional maturation. The DNMT1 gene encodes DNA methyltransferase 1, the primary enzyme responsible for maintaining DNA methylation patterns during cell division. COMMD1 is a negative regulator of NFκB-HIF signaling. These findings indicate that miR-145 upregulation in tumors is driven by both NOTCH signaling and promoter DNA hypomethylation.

## Discussion

This study integrates experimental and computational approaches to identify miR-145 as a key driver of carcinoma aggressiveness and the fibrotic tumor microenvironment subtype. A model is proposed in Figure 8. MiR-145 facilitates the fibrotic phenotype by activating heterotypic signaling pathways, including EMT, Angiogenesis, NOTCH, TGFB, HYPOXIA, Wnt/β-Catenin, Hedgehog and Myogenesis, while suppressing IFN-γ and JAK-STAT signaling. Elevated miR-145 is associated with CAF activation, extracellular matrix remodeling, and an immune-evasive TME. The data suggest that cancer–stromal juxtacrine interactions enhance NOTCH signaling in a positive feedback loop with miR-145, further reinforced by miR-145 promoter hypomethylation. Functionally, miR-145 overexpression induces autophagy and mitochondrial metabolism *in vitro*, and drives aggressive tumor growth *in vivo*, characterized by increased fibrosis, angiogenesis and reduced cytotoxic T-cell infiltration.

**FIGURE 8:**
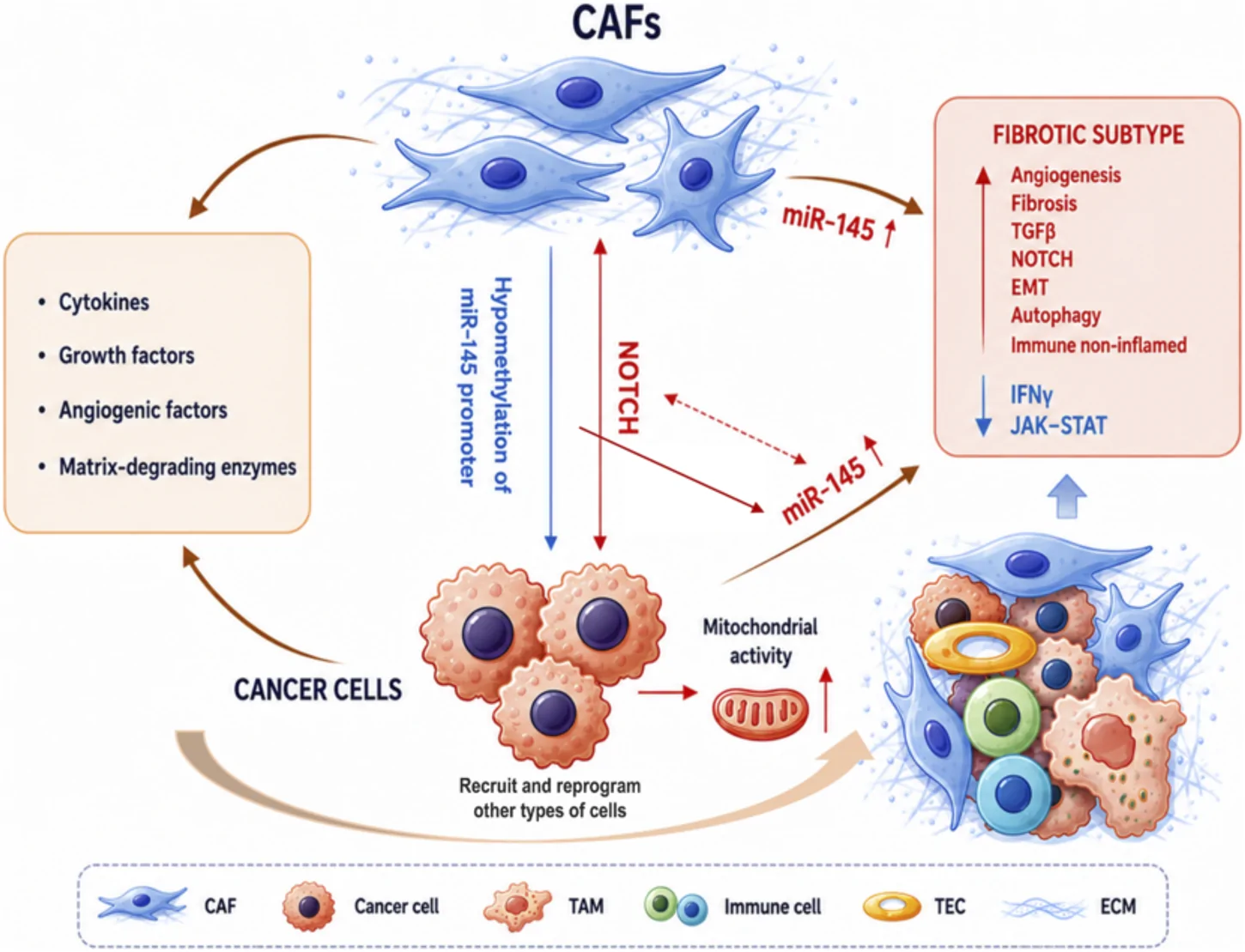
Model illustrating miR-145-mediated regulation of the TME.

MiR-145 is highly expressed in the tumor stromal compartment (Chivukula et al., 2014; Dimitrova et al., 2016), although most prior studies have focused on cancer cells. In human vascular smooth muscle cells, JAG1/NOTCH signaling induces miR-143/145 expression (Boucher et al., 2011), while p53 promotes miR-145 post-transcriptional maturation (Suzuki et al., 2009). Within the tumor microenvironment, paracrine and juxtacrine signaling coordinate communication between tumor and stromal cells. Paracrine factors such as cytokines, growth factors, chemokines, and TGF-β released by cancer cells and cancer-associated fibroblasts promote tumor growth, stemness, therapy resistance, immune evasion, and TME remodeling (Masih et al., 2022). Juxtacrine signaling requires direct cell-cell contact, with NOTCH signaling serving as a major communication pathway among cancer cells, CAFs, endothelial cells, immune cells, and tumor-associated macrophages (Lu et al., 2014; Parikainen et al., 2026). Other contact-dependent interactions, such as PD-L1/PD-1 signaling, also facilitate immune evasion (Yang et al., 2018). Cytonemes are specialized, actin-based membrane protrusions that mediate targeted, long-range cell-to-cell signaling. In cancer, tumor cells hijack cytonemes to establish direct communication with stromal cells by delivering growth factors, morphogen and receptor complexes that promote proliferation, invasion, and therapy resistance. Cytoneme biogenesis is initiated by morphogen–receptor signaling, including Hedgehog–Ihog and Wnt–Frizzled. Related membrane protrusions, such as tunneling nanotubes, further support tumor–stromal communication by transferring microRNAs, oncogenic proteins mutant KRAS, and even organelles, including mitochondria (Yamada et al., 2026; Ma et al., 2025). In addition, extracellular vesicle-mediated miRNA communication has emerged as a mechanism by which tumor and stromal cells remodel the TME, promoting cancer progression through enhanced angiogenesis, EMT, matrix remodeling, tumor stiffness and Immune evasion (Cocks et al., 2021; Sheta et al., 2023; Feng et al., 2026). Future studies will need to determine how crosstalk mediated by miR-145 occurs. Our data show that miR-145 induction *in vitro* depends on direct fibroblast-cancer cell contact and activation of membrane-bound NOTCH signaling. Notably, miR-145 also enhances NOTCH signaling, suggesting a reciprocal positive-feedback loop whose molecular basis remains to be elucidated.

Tumor angiogenesis is a hallmark of cancer progression, and the miR-143/145 cluster has emerged as an important regulator of neovascularization. Our data show that miR-145 promotes recruitment of CAFs, pericytes, and endothelial cells, remodels the extracellular matrix, enhances sprouting angiogenesis, and enriches EMT, NOTCH, hypoxia, and autophagy pathways within the tumor microenvironment. Tumor-associated angiogenesis arises from sprouting of existing vessels or differentiation of bone marrow- or tumor-derived stem cells, regulated by complex interactions among cells, ECM components, and physical factors such as pH, temperature, interstitial fluid pressure, and hypoxia (Korneev et al., 2017). CAFs further support angiogenesis through ECM remodeling and secretion of pro-angiogenic factors. Resistance to anti-angiogenic therapy commonly involves activation of NOTCH and hypoxia signaling, recruitment of bone marrow–derived cells, increased pericyte coverage, and enhanced invasion and metastasis to access normal vasculature (Bergers and Hanahan, 2008). Autophagy promotes tumor cell survival under stress (Sukhai et al., 2013; Zhitomi et al., 2016; Ibrahim et al., 2016) and can facilitate escape from anti-angiogenic treatment. Our data suggests that miR-145 induces autophagy, but future studies are required to determine conclusively its role on autophagy and whether tumors with high miR-145 expression may benefit from combined targeting of angiogenesis and autophagy.

Mitochondria play a central and multifunctional role in malignant tumor progression (Zong et al., 2016; Moreno-Sanchez et al., 2014). Mitochondrial oxidative phosphorylation supplies a substantial fraction of tumor ATP production (Dielschneider et al., 2017), and growing evidence links enhanced mitochondrial metabolism to metastatic potential. GAPDH as a classical cytosolic glycolytic enzyme can translocate to mitochondria under oxidative stress and apoptosis. Mitochondrial GAPDH regulates mitophagy, supports heme trafficking, and coordinates metabolic crosstalk with mitochondrial respiratory complexes (Nakajima et al., 2017; Tristan et al., 2011). Beyond its housekeeping role, GAPDH is a metabolic and oncogenic biomarker associated with aggressive disease and poor overall and relapse-free survival across multiple cancers, including lung, HNSCC, diffuse large B Cell lymphoma and gastrointestinal cancers (Révillion et al., 2000; Wiel et al., 2019; Peng et al., 2023; Liu et al., 2017; Chiche et al., 2019). Elevated GAPDH expression is also linked to a non-inflamed tumor microenvironment including fibrotic and immune-depleted subtypes characterized by reduced CD8^+^ T Cell infiltration, hypoxia-driven metabolic reprogramming, extracellular matrix remodeling, and immune exclusion, all of which promote tumor progression and therapy resistance (Peng et al., 2023; Liu et al., 2017; Chiche et al., 2019; Cohen et al., 2022). Collectively, our findings suggest that miR-145 promotes angiogenesis and mitochondrial activity, thereby enhancing tumor aggressiveness.

EMT promotes cancer immune evasion through multiple mechanisms. Mesenchymal-like cancer cells undergoing EMT drive the accumulation of Tregs and MDSCs, suppressing T Cell function. EMT transcription factors (Snail, Twist, and Zeb1) promote M2-like macrophage polarization and inhibit cytotoxic T lymphocytes through IL-4, IL-10, TGF-β, PD-L1, arginase I, and IDO-1. EMT also impairs antigen processing and presentation, further weakening anti-tumor immunity. While targeting EMT, its upstream regulators (e.g., TGF-β and Wnt), or epigenetic modulators (LSD1 and HDACs) can restore immune function, EMT reversal may also increase metastatic risk (Holmgaard et al., 2018; Yang et al., 2017; Duan et al., 2017). Consistent with these findings, our data clarify that miR-145 significantly upregulates EMT, TGF-β, and Wnt signaling pathways, contributing to immune evasion.

In summary, our findings identify miR-145 as a key driver of a fibrotic tumor microenvironment. miR-145 upregulation in cancer cells is promoted by promoter hypomethylation and activated NOTCH signaling. Functionally, miR-145 enhances autophagy and mitochondrial metabolism *in vitro*, while promoting tumor growth *in vivo* through coordinating EMT, angiogenesis, fibrosis, TGF-β signaling, and immune evasion characterized by reduced CD8^+^ T cell infiltration. Although functional validation using miR-145 knockdown or knockout models would provide additional mechanistic insights, miR-145 expression is markedly downregulated in tumor tissues compared with normal controls. Therefore, we focused on characterizing its baseline expression and functional associations across TME subtypes. This study identifies miR-145 as a potential biomarker of the fibrotic tumor microenvironment, providing a foundation for future mechanistic studies and therapeutic strategies.

## Data Availability

The original contributions presented in the study are included in the article/Supplementary Material, further inquiries can be directed to the corresponding author.
