# Supplementary material for: MiR-145 reprogramming facilitates fibrotic trajectory in the tumor microenvironment
Source: Front Cell Dev Biol. 2026 Oct 19;14:1850756. doi: 10.3389/fcell.2026.1850756 (PMC13737585; doi:10.3389/fcell.2026.1850756)
Supplement: Supplementary file 1 [file DataSheet1.pdf]

MiR-145 Reprogramming Facilitates Fibrotic Trajectory in the Tumor Microenvironment

Zhao Lin[1], Megan Bennett[1], Patrick Tassone[2], Joseph M. Curry[2],

and Ubaldo Martinez-Outschoorn[1,3]

1. Department of Medical Oncology, Sidney Kimmel Cancer Center, Thomas Jefferson University, Philadelphia, PA19107, USA.

2. Department of Otolaryngology, Sidney Kimmel Cancer Center, Thomas Jefferson University, Philadelphia, PA 19107, USA.

3. Correspondence should be addressed to U. M.-O (e mail: ubaldo.martinez-outschoorn@jefferson.edu)

Supplementary data

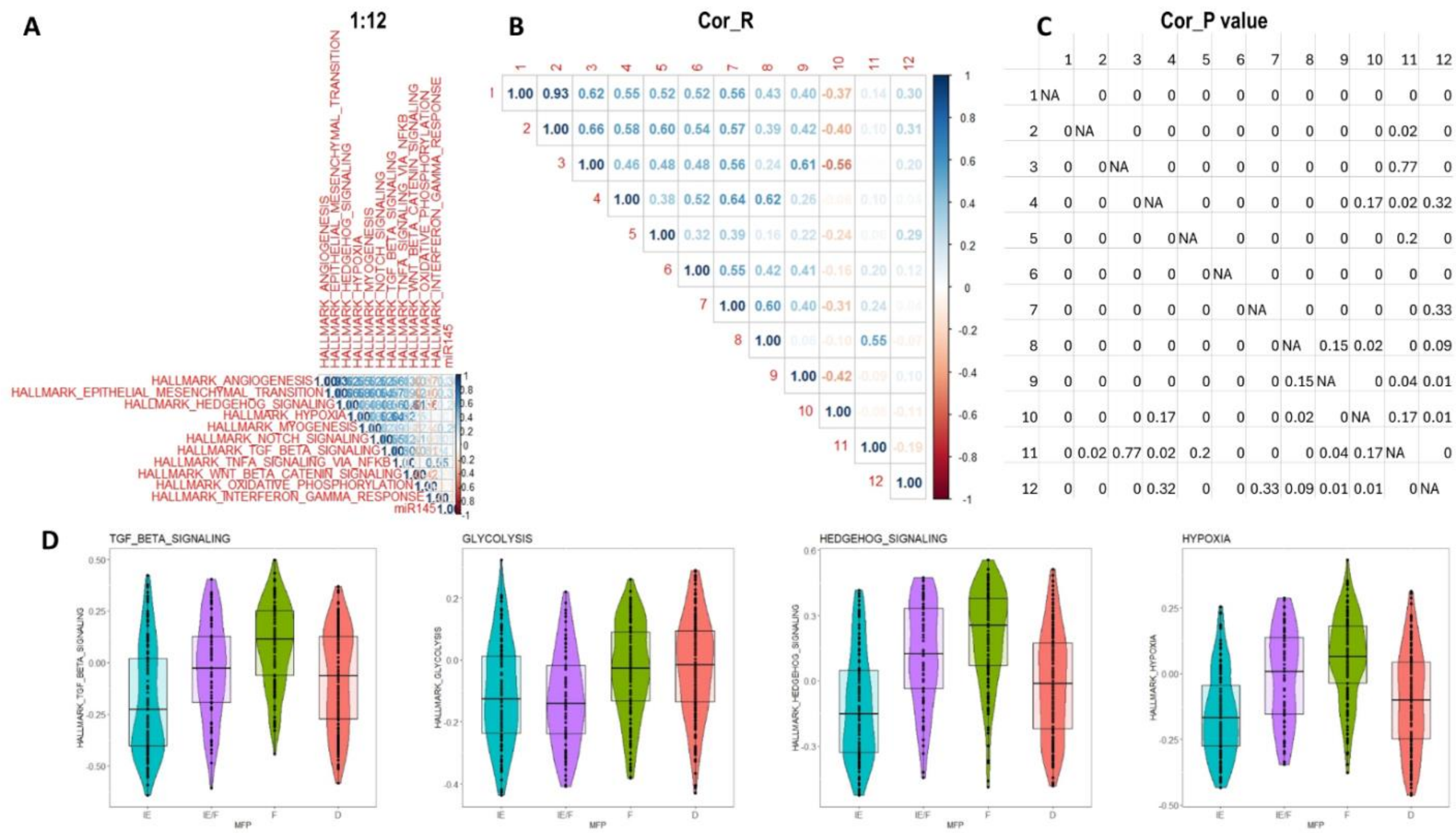

Supplementary Figure 1. The correlation of miR-145 expression with enrichment of Hallmark pathways in human HNSCC.

(A)-(D) miRNAseq and rnaSeq data from TCGA HNSC database was retrieved and classified as four distinct TME subtypes: IE, IE/F, F and D. (A)-(C) The correlation of miR-145 expression with enrichment of Hallmark pathways. (D) Violin plot showing the enrichment of hypoxia, TGFB, glycolysis, HEDGEHOG pathways in the four TME subtypes.

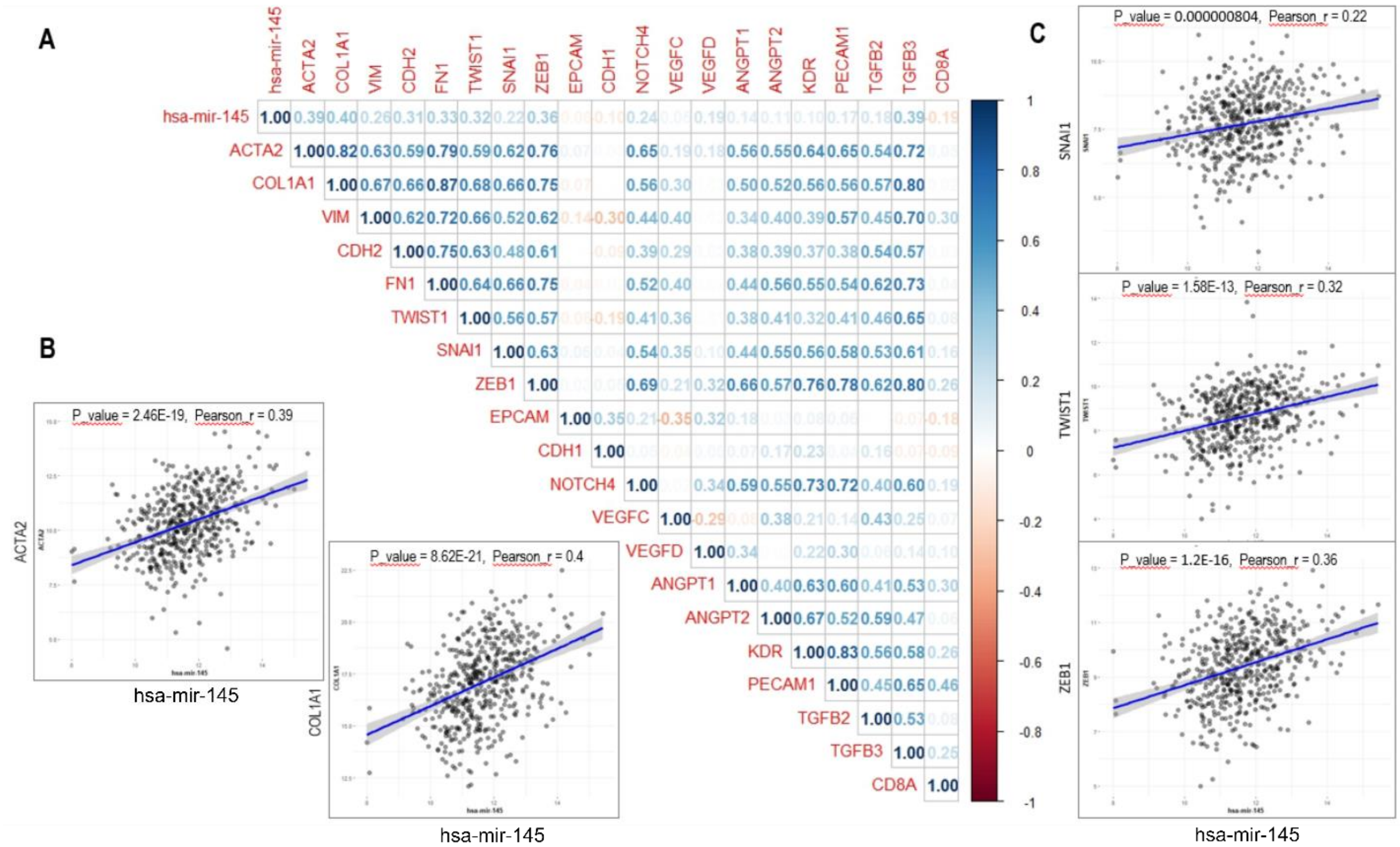

Supplementary Figure 2. miR-145 expression correlated with a panel of genes linking to diverse TME signaling pathways including EMT, CAFs, angiogenesis and TGF-β.

(A)-(C) miRNAseq and rnaSeq data from TCGA HNSCC database was retrieved.

(A) Correlation of miR-145 expression with multiple genes.

(B) Correlation of miR-145 expression with CAFs: ACTA2, COL1A1.

(C) Correlation of miR-145 expression with EMT_TFs: SNAI1, TWIST1, ZEB1.

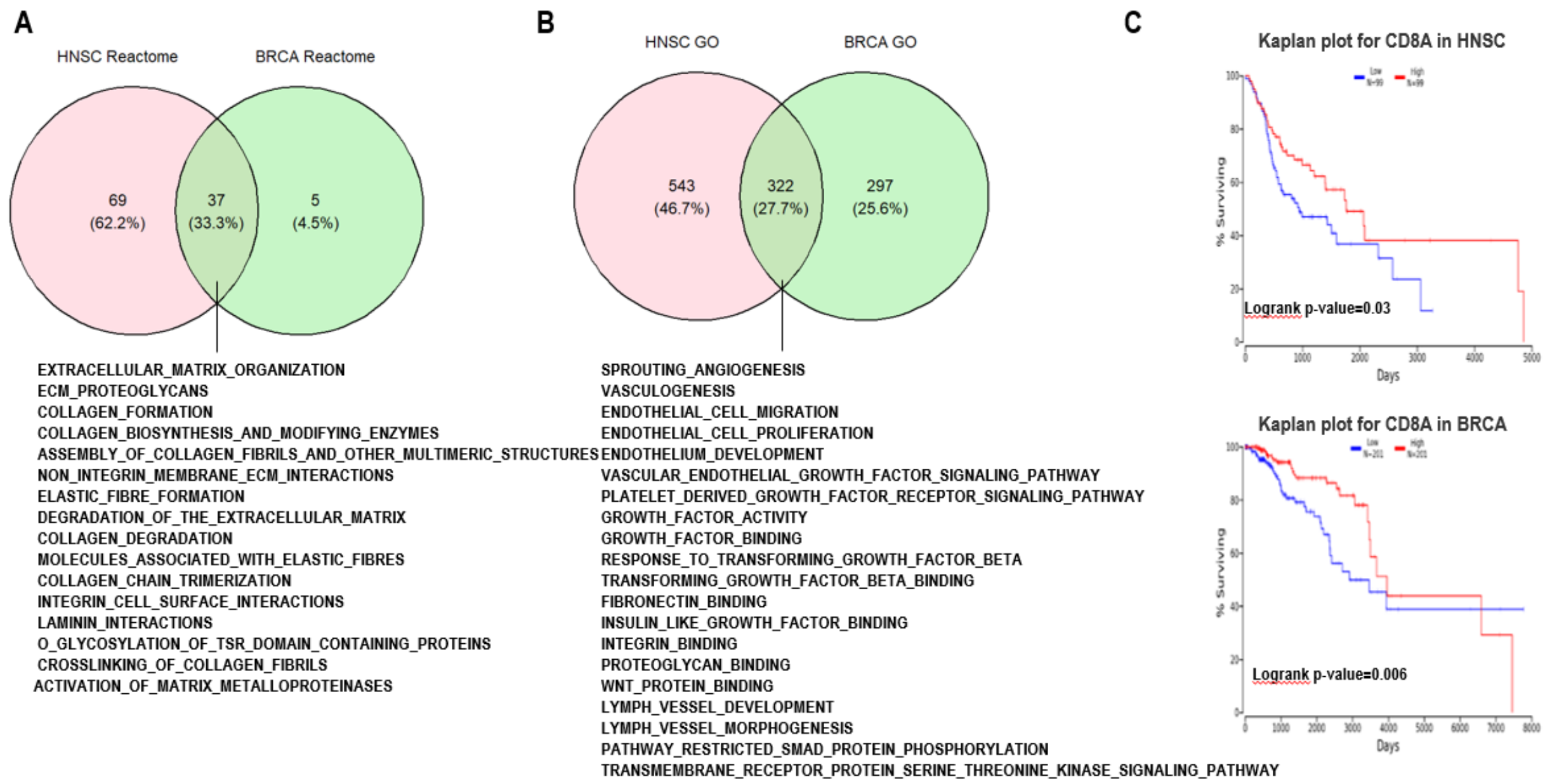

Supplementary Figure 3. High miR-145 is associated with tumor angiogenesis accompanied by Reactome pathways and Gene Ontology changes in human HNSCC and BRCA.

(A)-(B) miRNAseq and rnaSeq data from HNSCC and BRCA TCGA database was classified as top 25% high and low miR-145 group. (A)Venn diagram of upregulated Reactome pathways. (B)Venn diagram of Gene Ontology.

(C) Overall survival Kaplan plot for CD8A in Oncolnc.

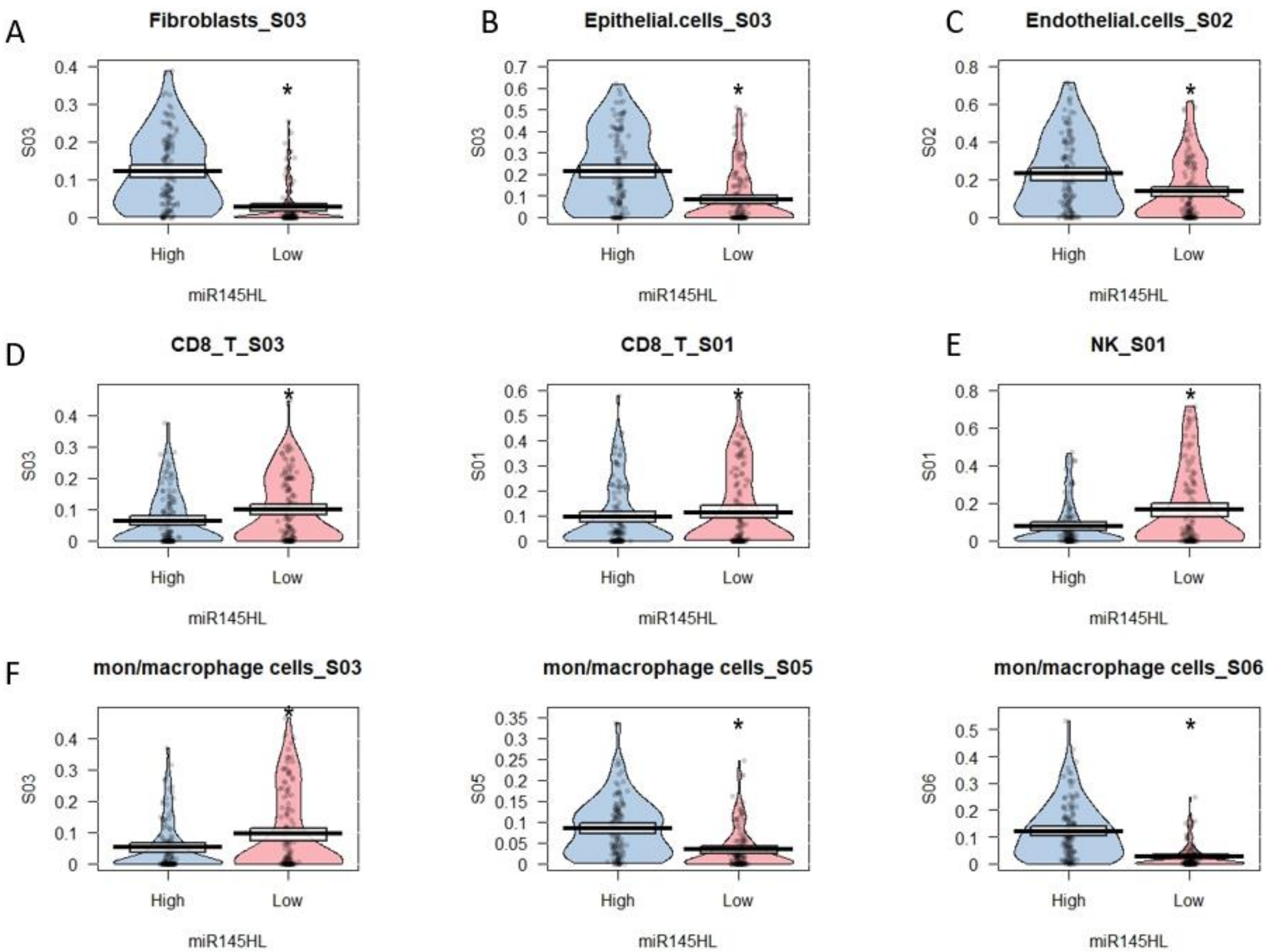

Supplementary Figure 4. High miR-145 defined subset of cell states in human HNSCC.

(A)-(F) miRNAseq and RNAseq data from HNSCC TCGA database was classified as top 25% high and low miR145 group. Ecotyper carcinoma cell state abundance was carried out. Displays of high versus low groups. p adjusted < 0.05.

(A) Fibroblasts state S03. (B) Epithelial cells state S03. (C) Endothelial cells state S02. (D) CD8 T state S01, and S03. (E) NK state S01.

(F) monocyte/macrophage cells state S03, S05, and S06.

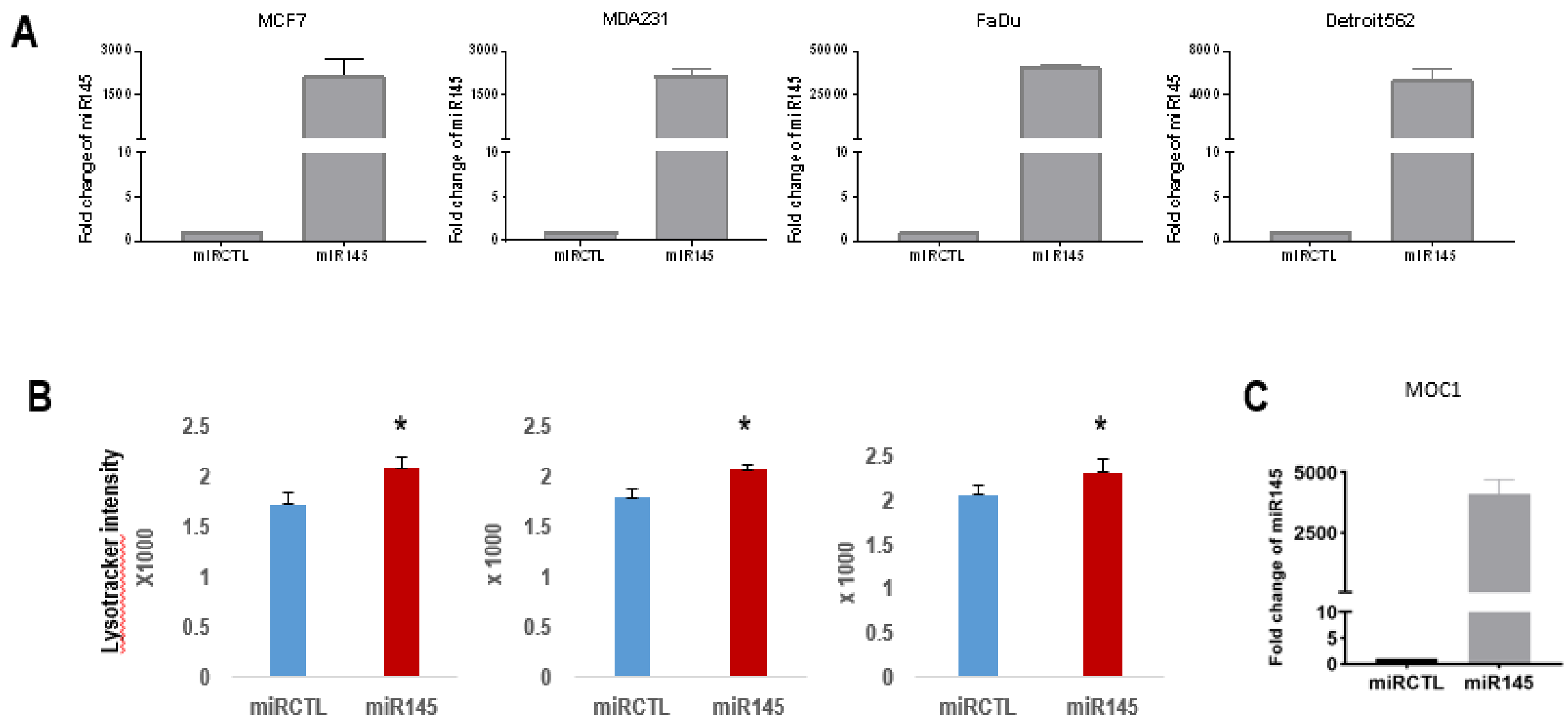

Supplementary Figure 5. MiR-145 overexpression and its effects on lysosomal activity.

(A)Bar plots showing fold change of miR-145 in MCF7, MDA231, FaDu and Detroit562 cells expressing precursor miR-145 and scramble control.

(B)Flow plots showing lysotracker level in different cancer cells expressing precursor miR-145 and scramble control.

(C)Bar plot showing fold change of miR-145 in MOC1 cells expressing precursor miR-145 and scramble control. (n=3, asterisk indicates P<0.05). Error bars denote ±SD.

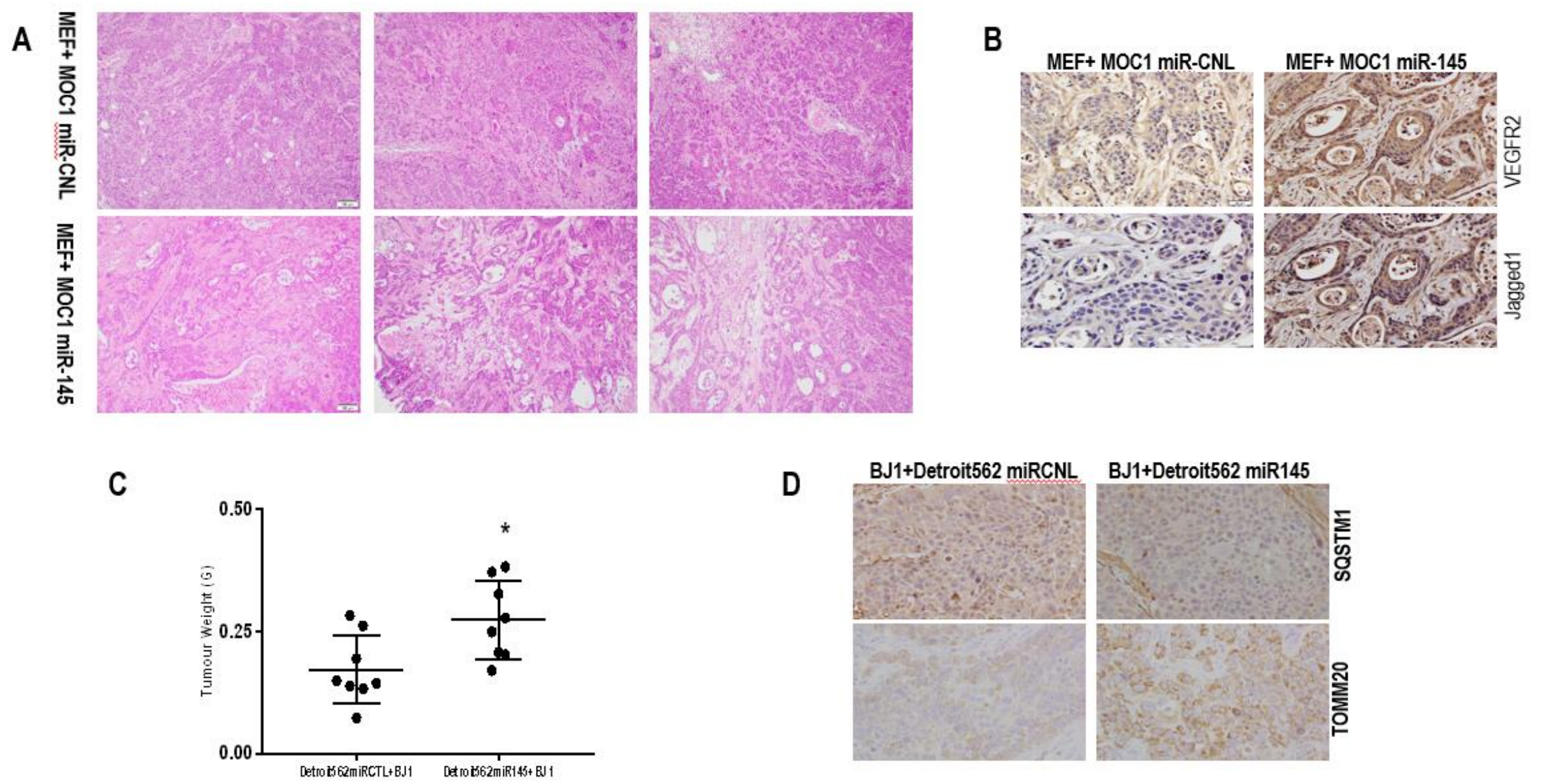

Supplementary Figure 6. MiR-145 in cancer cells upregulated angiogenesis coupled with autophagy.

(A-B) MEF cells and MOC1 cells overexpressing miR-145 and scramble control were injected into mice. Tumor tissues were processed at the end point of 4 weeks post-injection after resection. (A) Representative H&E staining of tumor tissues. (B) IHC for angiogenesis drivers and markers VEGFR2 and Jagged1.

(C-D) BJ1 fibroblast cells and Detroit562 cells overexpressing miR-145 and scramble control were coinjected into mice. (C)Tumor weight was measured at end point. (n=10, asterisk indicates P<0.05). Error bars denote ±SD. (D) IHC staining for autophagy marker SQSTM1 and mitochondrial metabolism driver and marker TOMM20.

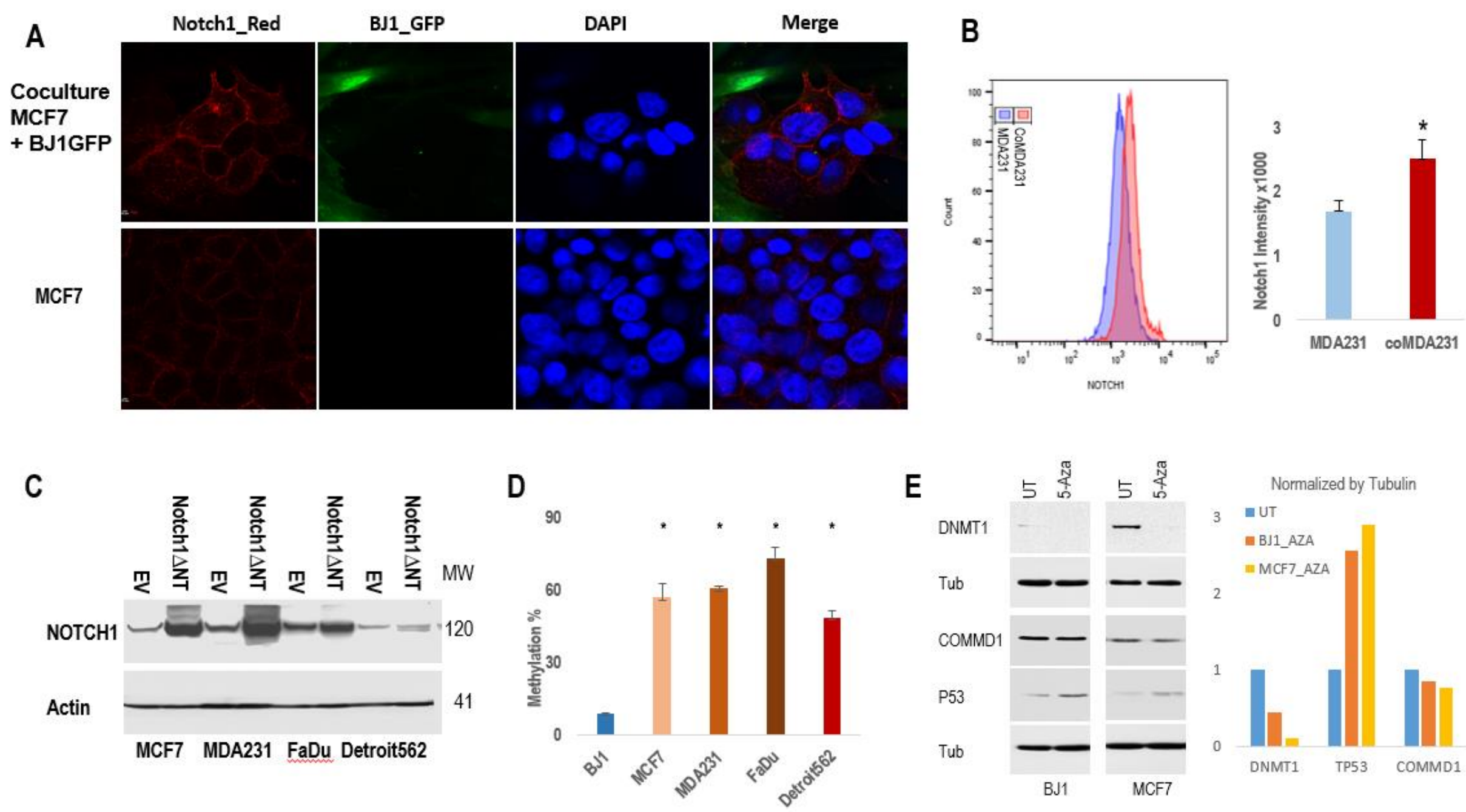

Supplementary Figure 7. The expression of NOTCH1 and methylation in different cells.

(A) Images of NOTCH1 (Red) expression in MCF7 cancer cells under coculture with BJ1GFP or homotypic culture condition (scale bar: 20 μm).

(B) Flow plots showing NOTCH1 level in MDA-MB231 cancer cells with coculture and homotypic culture. (n=3, asterisk indicates $p<0.05$). Error bars denote ±SD.

(C) NOTCH1 expression in different cancer cells overexpressing NOTCH1ΔNT or empty vector control.

(D) The level of DNA methylation in BJ1 fibroblast cells and various cancer cells.

(E)Western Blot showed that 5-Aza suppressed DNMT1 and COMMD1, induced TP53 expression in hTert-BJ1 and MCF7 cells.
